# Assessing the impact of the Global Point Prevalence Survey of Antimicrobial Consumption and Resistance (Global-PPS) on hospital antimicrobial stewardship programmes: results of a worldwide survey

**DOI:** 10.1186/s13756-021-01010-w

**Published:** 2021-09-28

**Authors:** Ines Pauwels, Ann Versporten, Helene Vermeulen, Erika Vlieghe, Herman Goossens

**Affiliations:** 1 Laboratory of Medical Microbiology, Vaccine & Infectious Disease Institute (VAXINFECTIO), Faculty of Medicine and Health Sciences, University of Antwerp, Antwerp, Belgium; 2 Interuniversity Institute for Biostatistics and Statistical Bioinformatics (I-BIOSTAT), Data Science Institute, Hasselt University, Diepenbeek, Belgium; 3 Department of General Internal Medicine, Infectious Diseases and Tropical Medicine, University Hospital Antwerp, Antwerp, Belgium; 4 Global Health Institute, Faculty of Medicine and Health Sciences, University of Antwerp, Antwerp, Belgium

**Keywords:** Hospital, Antimicrobial stewardship, Point prevalence survey

## Abstract

**Background:**

The Global Point Prevalence Survey of Antimicrobial Consumption and Resistance (Global-PPS) provides a methodology to support hospitals worldwide in collecting antimicrobial use data. We aim to evaluate the impact of the Global-PPS on local antimicrobial stewardship (AMS) programmes and assess health care professionals’ educational needs and barriers for implementing AMS.

**Methods:**

A cross-sectional survey was disseminated within the Global-PPS network. The target audience consisted of hospital healthcare workers, involved in local surveillance of antimicrobial consumption and resistance. This included contacts from hospitals that already participated in the Global-PPS or were planning to do so. The survey contained 24 questions that addressed the hospital’s AMS activities, experiences conducting the PPS, as well as the learning needs and barriers for implementing AMS.

**Results:**

A total of 248 hospitals from 74 countries participated in the survey, of which 192 had already conducted the PPS at least once. The survey response rate was estimated at 25%. In 96.9% of these 192 hospitals, Global-PPS participation had led to the identification of problems related to antimicrobial prescribing. In 69.3% at least one of the hospital’s AMS components was initiated as a result of Global-PPS findings. The level of AMS implementation varied across regions. Up to 43.1% of all hospitals had a formal antimicrobial stewardship strategy, ranging from 10.8% in Africa to 60.9% in Northern America. Learning needs of hospitals in high-income countries and in low-and middle-income countries were largely similar and included general topics (e.g. ‘optimising antibiotic treatment’), but also PPS-related topics (e.g. ‘translating PPS results into meaningful interventions’). The main barriers to implementing AMS programmes were a lack of time (52.7%), knowledge on good prescribing practices (42.0%), and dedicated funding (39.9%). Hospitals in LMIC more often reported unavailability of prescribing guidelines, insufficient laboratory capacity and suboptimal use of the available laboratory services.

**Conclusions:**

Although we observed substantial variation in the level of AMS implementation across regions, the Global-PPS has been very useful in informing stewardship activities in many participating hospitals. More is still to be gained in guiding hospitals to integrate the PPS throughout AMS activities, building on existing structures and processes.

**Supplementary Information:**

The online version contains supplementary material available at 10.1186/s13756-021-01010-w.

## Background

Optimising the use of antimicrobial agents is a key element in the global response to the antimicrobial resistance (AMR) crisis [1]. The principles of antimicrobial stewardship (AMS) are increasingly being adopted, at organisational and national levels [2, 3]. Hospital AMS interventions have been shown to increase appropriate use of antibiotics, reduce treatment costs, resistance rates and healthcare-associated infections, and improve patient outcomes [4, 5]. Many hospitals worldwide are in different stages of implementing AMS activities. However, in low- and middle-income countries (LMIC) this has proven to be challenging due to a high infectious disease burden, limited access to certain antibiotics, unregulated use of antibiotics in the community and a lack of diagnostic capacity to guide clinical decision-making [6–11]. These hospitals are faced with an urgent need to set up locally-adapted, sustainable and scalable interventions to contain the problem of AMR. This requires robust, yet feasible, methods for monitoring antimicrobial use and resistance as these are among the cornerstones to a successful implementation and evaluation of antimicrobial stewardship programmes [9, 12, 13]. Point prevalence surveys (PPS) are commonly used within stewardship programmes to assess the quality of antimicrobial prescribing at ward- and institutional level and to inform local stewardship activities [14–16]. The Global Point Prevalence Survey of Antimicrobial Consumption and Resistance (Global-PPS), developed at the University of Antwerp, Belgium, provides a standardised method for assessing hospital antimicrobial prescribing, and supports participating hospitals in electronic data entry, validation and reporting of results [17]. Launched worldwide in 2015, repeated in 2017, and available on a four-monthly basis since 2018, the Global-PPS had been used in more than 700 different hospitals from over 70 different countries worldwide at the end of 2018, including many in LMIC [18]. While many of these hospitals have successfully gathered antimicrobial use data through the Global-PPS, it remains to be investigated how they are using these findings to inform contextualised stewardship activities. This paper reports the results of a cross-sectional survey sent out to hospitals in the Global-PPS network and aims to: (I) evaluate experiences from hospitals participating in the Global-PPS and assess its role in informing hospital AMS programmes, (II) identify barriers to implementing AMS in different resource settings, (III) explore the learning needs of healthcare workers involved in stewardship worldwide in terms of AMS and using the PPS to support hospital AMS programmes.

## Methods

### Setting and participants

Since 2015 the Global-PPS supports hospitals in gathering antimicrobial prescribing data on inpatient wards using the methodology of a point prevalence survey [17, 19]. These data include basic antimicrobial prescription data as well as a set of quality indicators for prescribing. After electronic data entry and validation, a real-time, personalised feedback report in pdf-format presents the hospitals’ PPS results in a series of graphs and tables. The Global-PPS resources are available at no cost and participation is voluntary and open to all hospitals worldwide. The Global-PPS methodology has been described in detail elsewhere [19]. In addition to collecting baseline antimicrobial prescribing data, hospitals can follow up on their stewardship interventions using repeated point prevalence surveys. Within the activities of the Global-PPS project, a self-administered, cross-sectional survey on antimicrobial stewardship was disseminated in the Global-PPS network. The respondents of the survey were local healthcare professionals, involved in surveillance of antimicrobial consumption and resistance, who had already conducted the Global-PPS in their hospital or were planning to do so.

### Survey development

A first set of questions addressed the structure and components of hospital AMS programmes before and after participation in the Global-PPS. These questions were developed based on surveys in existing literature and published standards and core elements for hospital AMS programmes [10, 12, 20–23]. In a second series of questions, focused on the Global-PPS automated feedback function, respondents were asked to comment on the comprehensiveness and usefulness of the report and to list the most important findings identified from this report in their respective institutions. The specific terminology used in these questions was adapted to the terminology used in the feedback report. Finally, the questionnaire also addressed barriers to implementation of AMS and specific learning needs of local stewardship teams on the topic of AMS. The respondents planning to conduct their first PPS were asked to only complete the questions on AMS activities, barriers and learning needs, as the Global-PPS-specific questions did not apply to them. The questionnaire was pilot-tested and reviewed for content and comprehensiveness by Global-PPS participants and experts in the field of AMS from different countries. The final version of the questionnaire contained a total of 24 questions and was translated into Arabic, Russian, French and Spanish (the full questionnaire is available in Additional file 1). We used Qualtrics (Provo, Utah, USA) to programme the electronic questionnaire and question logic was applied to reduce the time needed to complete the questionnaire and improve the survey completion rate.

### Data collection

The link to the online questionnaire was sent out by e-mail to the entire Global-PPS network. This included focal persons at individual hospital level as well as contacts responsible for a network of hospitals and country coordinators. Respondents working in multiple hospitals were asked to fill out one survey for the hospital which they considered most representative of their daily practice and to share the survey with their contacts working in other hospitals. We asked respondents to complete only one survey per hospital but did not restrict duplicate entries. Respondents had the option to fill in the survey through a personal invitation over email or through an anonymous survey link. Answers from respondents using the personal invitation were kept strictly confidential and were de-identified before data analysis. Responses were collected between February 2019 and May 2019 and reminders were sent 4 weeks, 9 weeks and 13 weeks after the initial invitation.

### Data analysis

Partially completed responses were only included if at least the question on hospital AMS components was answered. During data cleaning, free-text answers to multiple choice questions with an ‘Other, please specify response’ were re-categorised into one of the listed response options where applicable. Duplicate entries at institutional level were amalgamated into one response. As the survey was disseminated within national and regional networks, the final number of hospitals receiving the survey could not be identified, and therefore it was impossible to calculate an exact response rate. A response rate was therefore approximated based on the number of hospitals that had participated in the Global-PPS by the time the survey had closed. The hospitals stating that they did not yet conduct the Global-PPS were not included in this estimation. During data analysis, countries were grouped according to the 2019 World Bank income classification [24]. Categorical variables were analysed descriptively and results were presented as frequencies and percentages. As not all respondents replied to all survey questions, denominators are reported for every question separately. The differences between high-income countries and low- and middle- income countries for the AMS learning needs and barriers for implementation were evaluated using the Pearson’s chi-squared test or Fisher’s exact test as appropriate. Significance levels were corrected for multiple comparisons. Statistical analyses were performed using R version 3.6.1 (R Foundation for Statistical Computing, Vienna, Austria) with RStudio (RStudio, PBC, Boston, MA, USA).

## Results

### General results

By the end of the survey period, a total of 297 respondents had returned the questionnaire. Up to 23 records did not contain any information on hospital AMS structures and were excluded from the analyses. Another 26 duplicate records at institutional level had to be amalgamated. This finally resulted in 248 records to be included in the analyses. Of all hospitals returning the questionnaire, 77.4% (192/248) had participated at least once in the Global-PPS at the time of the survey. As a total of 765 hospitals had collected data for the Global-PPS at that time, the approximate response rate was estimated at 25% (192/765).

**Fig. 1:**
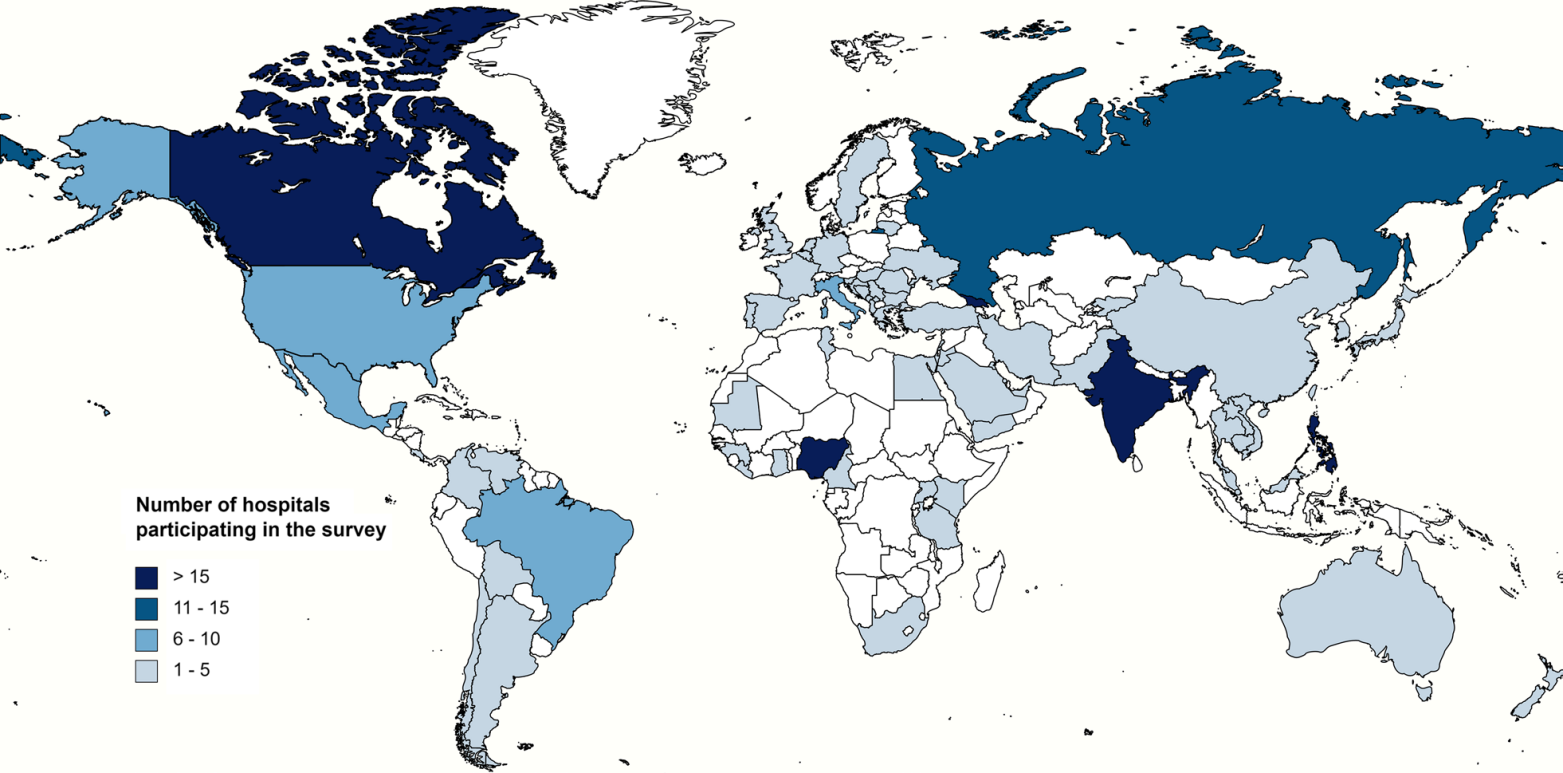
Overview of countries participating in the antimicrobial stewardship survey

Survey respondents consisted primarily of infectious diseases specialists (22.6%), pharmacists (20.1%), medical microbiologists (16.4%), clinicians (15.3%), and infection prevention and control (IPC) specialists (12.4%). The 248 included hospitals were from 74 different countries (Fig. 1): 91 (36.7%) from Asia, 61 (24.6%) from Europe, 38 (15.3%) from Africa, 31 (12.5%) from Latin America and the Caribbean, 24 (9.7%) from Northern America, and 3 (1.2%) from Oceania. High-income countries represented 33.1% (82/248) of all responses, whereas upper middle-income and lower middle-income countries accounted for 35.1% (87/248) and 28.2% (70/248), respectively. There were 9 (3.6%) responses from low-income countries. A detailed overview of participating countries is available in Additional file 2. Up to 93.5% (232/248) of hospitals returned a fully completed questionnaire. The majority of hospitals were tertiary hospitals (65.7%, 163/248) (Table 1; hospital characteristics stratified by region are available in Additional file 3).

**Table 1:** Hospital characteristics

	n (%)		
	Hospitals that conducted PPS (n = 192)	Hospitals planning to conduct PPS (n = 56)	Total number of hospitals (n = 248)
Hospital type*			
Tertiary hospital	134 (69.8)	29 (51.8)	163 (65.7)
Secondary hospital	28 (14.6)	16 (28.6)	44 (17.7)
Paediatric hospital	12 (6.3)	1 (1.8)	13 (5.2)
Other specialised hospital	7 (3.6)	5 (8.9)	12 (4.8)
Primary care institution	6 (3.1)	5 (8.9)	11 (4.4)
Infectious diseases specialised hospital	5 (2.6)	0 (0.0)	5 (2.0)
*Teaching hospital*			
Yes	153 (79.7)	44 (78.6)	197 (79.4)
No	39 (20.3)	12 (21.4)	51 (20.6)
Number of inpatient beds**			
Less than 100	20 (10.4)	8 (14.3)	28 (11.3)
101–250	43 (22.4)	14 (25.0)	57 (23.0)
251–500	59 (30.7)	17 (30.4)	76 (30.6)
501–1000	43 (22.4)	10 (17.9)	53 (21.4)
1001–2000	20 (10.4)	5 (8.9)	25 (10.1)
More than 2000	7 (3.6)	2 (3.6)	9 (3.6)

*Tertiary hospital: clinical services are highly differentiated by function. Provides regional services and regularly takes referrals from other (primary and secondary) hospitals. Secondary hospital: clinical services are highly differentiated by function. Takes some referrals from other (primary) hospitals. Primary care institution: has only few medical specialties. Only limited laboratory services are available. Infectious diseases specialised hospital and paediatric hospital: single clinical specialty, possibly with sub-specialties. Highly specialised staff and technical equipment [25]. **Inpatient beds: accommodate hospitalized patients who stay in the hospital for a minimum of one night

### Global-PPS experiences

**Fig. 2:**
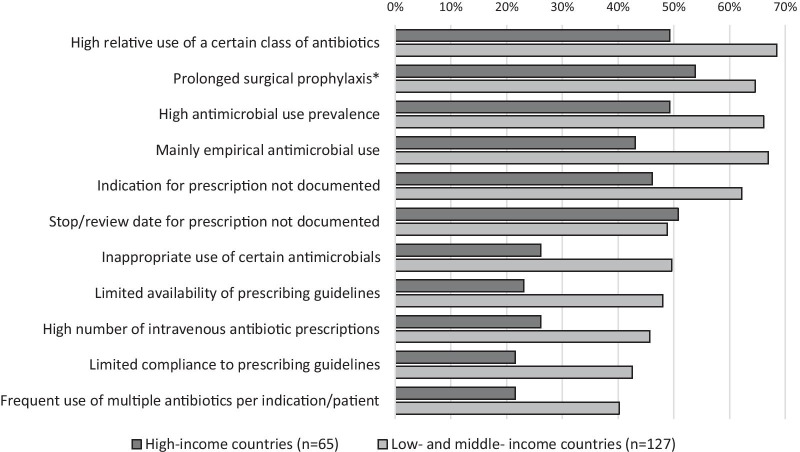
Problems related to antimicrobial prescribing as identified from Global-PPS results (% of hospitals). *Perioperative antibiotics for prevention of surgical site infections, administered for a period > 24 h

Of the 192 hospitals that participated at least once in the Global-PPS, 81.8% (157/192) stated that they used the personalised feedback report, available after validation of the hospital’s PPS data. In 96.9% (186/192) of all hospitals, at least one observation related to antimicrobial prescribing, which could be a target for improvement, was identified from initial PPS findings. The most common prescription-related problems were a high relative use of a certain class of antibiotics (62.0%; 119/192), prolonged surgical antibiotic prophylaxis (60.9%; 117/192), and a high antimicrobial use prevalence (60.4%; 116/192) (Fig. 2). Of all hospitals participating in the Global-PPS, 33.2% (63/190) reported that they conducted a follow-up PPS, to assess the impact of their stewardship activities on hospital antimicrobial prescribing, and another 40% (76/190) planned to do so. Up to 85.7% (54/63) of the hospitals that conducted a follow-up PPS stated that they observed an improvement in one or more of the prescribing-related problems identified earlier. About half (50.8%; 32/63) observed a decrease in antimicrobial use prevalence, 47.6% (30/63) could see an improvement in the documentation of the reason for prescription, and 46.0% (29/63) observed a decrease in the relative use of a certain class of antibiotics. Just over half of the hospitals that conducted the PPS (54.0%; 94/174) considered it feasible to repeat a hospital-wide PPS on a yearly basis, while 24.7% (43/174) would repeat the PPS every 2 years and 20.7% (36/174) considered doing a hospital-wide PPS 2 or 3 times per year. The main motivations for performing repeated PPS’ were to create awareness on appropriate antimicrobial prescribing (75.4%; 138/183) and to continuously monitor the quality and quantity of antimicrobial prescriptions (76.0%; 139/183).

### AMS structures and interventions

Up to 43.1% (100/232) of all hospitals reported the presence of a formal antimicrobial stewardship strategy, defined as a plan that describes the aims, milestones and outcome measures of stewardship activities. This ranged from 10.8% (4/37) in Africa to 60.9% (14/23) in Northern America. Another 29.7% (69/232) reported that the development of such an antimicrobial stewardship strategy was planned. The availability of local, evidence-based prescribing guidelines was substantially lower in African hospitals compared to other regions (Table 2). Northern American hospitals scored particularly high for the presence of guidelines, AMS committees and specific stewardship interventions. Of all hospitals participating in the Global-PPS and with AMS activities, 69.3% (124/179) stated that at least one of these activities had been initiated as a result of PPS findings. The Global-PPS was mostly used to inform education and communication and to inform the development and review of guidelines (Table 2). A comparison of the AMS structures in hospitals that had already participated in the Global-PPS and those hospitals that did not yet participate can be found in Additional file 4.

**Table 2:** Hospital AMS structures and activities, overall and by region

	n (%)						n/n (%)
	Africa (n = 38)	Asia (n = 91)	Europe (n = 61)	Latin America (n = 31)	Northern America (n = 24)	Total (n = 248)	Initiated as a result of PPS findings*
Local, evidence-based guidelines	12 (31.6)	69 (75.8)	44 (72.1)	24 (77.4)	23 (95.8)	175 (70.6)	67/143 (46.9)
Antimicrobial formulary	9 (23.7)	68 (74.7)	41 (67.2)	14 (45.2)	21 (87.5)	156 (62.9)	42/126 (33.3)
Education and communication	15 (39.5)	38 (41.8)	40 (65.6)	19 (61.3)	16 (66.7)	129 (52.0)	50/98 (51.0)
AMS committee**	12 (31.6)	46 (50.5)	27 (44.3)	16 (51.6)	23 (95.8)	127 (51.2)	34/102 (33.3)
AMS team^†^	8 (21.1)	42 (46.2)	31( 50.8)	16 (51.6)	19 (79.2)	119 (48.0)	27/95 (28.4)
Specific AMS interventions^††^	7 (18.4)	31 (34.1)	24 (39.3)	11 (35.5)	23 (95.8)	99 (39.9)	32/81 (39.5)
Information technology support	1 (2.6)	43 (47.3)	14 (23.0)	7 (22.6)	9 (37.5)	76 (30.6)	25/62 (40.3)
Other AMS activities	1 (2.6)	3 (3.3)	2 (3.3)	2 (6.5)	2 (8.3)	10 (4.0)	
No AMS activities	11 (28.9)	6 (6.6)	1 (1.6)	2 (6.5)	0 (0.0)	20 (8.1)	

Results for Oceania (n = 3) are not reported separately

*For the group of hospitals participating in the Global-PPS and with the respective AMS component implemented in the hospital

**The organizational structure responsible for defining the antimicrobial stewardship strategy [12]

^†^The core operational team, responsible for the implementation of the antimicrobial stewardship activities in daily practice [12]

^††^E.g. audit and feedback, automatic stop orders, intravenous-to-oral switch policies etc.…

### Education

Up to 86.4% (210/243) of hospitals stated that they educated clinicians on appropriate prescribing, ranging from 71.1% in Africa to 93.4% in Europe. In 66.3% (161/243) of all hospitals, clinicians received written materials, such as leaflets, guideline booklets or newsflashes. Education for clinicians in the format of on-the-job-training and occasional, short training sessions was organised in 51.4% (125/243) and 49.8% (121/243) of hospitals, respectively. Nurses received training on antimicrobial stewardship in 65.4% (159/243) of hospitals (written information: 36.6%; on-the-job-training: 32.9%; occasional training sessions: 29.6%). The number of hospitals that delivered training to nurses was particularly high in Northern America (73.9%) and Asia (75.0%). In 55.6% (135/243) of hospitals, education delivery was foreseen for pharmacists, ranging from 37.7% in Europe to 82.6% in Northern America (written information: 37.0%; occasional training sessions: 25.1%; on-the-job-training: 22.6%).

We subsequently asked hospitals which educational activities on AMS would support them in continuing their stewardship efforts, and 76.3% (177/232) replied that they would benefit from face-to-face training sessions from stewardship champions. Up to 73.3% (170/232) considered case-based learning useful, and over half of the hospitals (57.8%; 134/232) stated that they would like to engage in joint research activities on the implementation of AMS. From a list of potential topics for learning activities, hospitals were asked to identify the topics they considered most useful (Table 3). These topics were largely similar for hospitals in high-income countries and those in low-and middle-income countries.

**Table 3:** Learning needs of hospitals on AMS

	n (%)			
	High-income countries (n = 76)	Low- and middle-income countries (n = 156)	Total (n = 232)	*P*-value^†^ (α = 0.0036)
Optimising therapeutic antimicrobial use	43 (56.6)	105 (67.3)	148 (63.8)	0.147
Optimising surgical prophylaxis	38 (50.0)	91 (58.3)	129 (55.6)	0.290
Translating PPS results into AMS interventions	38 (50.0)	71 (45.5)	109 (47.0)	0.615
Communicating with prescribers	35 (46.1)	61 (39.1)	96 (41.4)	0.386
Managing difficult-to-treat MDRO infections*	25 (32.9)	71 (45.5)	96 (41.4)	0.091
Identifying the low-hanging fruit for AMS in the hospital	38 (50.0)	56 (35.9)	94 (40.5)	0.056
Translating PPS results into IPC** interventions	17 (22.4)	61 (39.1)	78 (33.6)	0.017
Formulating/revising guidelines	21 (27.6)	54 (34.6)	75 (32.3)	0.359
Performing audit and feedback	17 (22.4)	54 (34.6)	71 (30.6)	0.081
Understanding antimicrobial susceptibility data	15 (19.7)	55 (35.3)	70 (30.2)	0.024
How to create an active stewardship committee/team	9 (11.8)	41 (26.3)	50 (21.6)	0.019
How to communicate with patients on antimicrobial use	13 (17.1)	28 (18.0)	41 (17.7)	1.000
Other learning needs	1 (1.3)	2 (1.3)	3 (1.3)	1.000
None	2 (2.6)	0 (0.0)	2 (0.9)	0.106

*MDRO: multi-drug resistant organisms; **IPC: infection prevention and control †Statistical significance evaluated using the Pearson’s chi-squared test or Fisher’s exact test. Significance level (α) has been corrected for multiple testing

### Barriers to implementing AMS

Globally, the main barriers to implementing hospital AMS programmes were a lack of time to work on AMS activities (52.7%; 128/243), a lack of knowledge on good prescribing practices (42.0%; 102/243), and a lack of dedicated funding for the AMS programme (39.9%; 97/243). Although there was a certain degree of similarity in the barriers identified in high-income countries and low- and middle-income countries, significant differences could be observed for certain barriers (Table 4). Hospitals in low- and middle-income countries were more often confronted with unavailability of prescribing guidelines (35.6% vs. 7.5%; *p* < 0.001), insufficient laboratory capacity (35.0% vs. 12.5%; *p* < 0.001) and suboptimal use of the available laboratory services (21.5% vs. 2.5%; *p* < 0.001). In high-income countries, a lack of information technology to support antimicrobial prescribing was more frequently identified as a barrier, compared to low-and middle-income countries (46.3% vs. 22.1%; *p* < 0.001).

**Table 4:** Barriers to implementation of AMS

	n (%)			
	High-income countries (n = 80)	Low- and middle-income countries (n = 163)	Total (n = 243)	*p*-value* (α = 0.0026)
Lack of time to perform AMS activities	50 (62.5)	78 (47.9)	128 (52.7)	0.044
Lack of knowledge on good prescribing practices	28 (35.0)	74 (45.4)	102 (42.0)	0.160
Lack of funding for AMS programme	41 (51.3)	56 (34.4)	97 (39.9)	0.017
Lack of cooperation from prescribers	21 (26.3)	67 (41.1)	88 (36.2)	0.034
Lack of information technology	37 (46.3)	36 (22.1)	73 (30.0)	** < 0.001**
Unavailability of prescribing guidelines	6 (7.5)	58 (35.6)	64 (26.3)	** < 0.001**
Lack of qualified personnel	13 (16.3)	44 (27.0)	57 (23.5)	0.090
Lack of support from hospital management	14 (17.5)	40 (24.5)	54 (22.2)	0.282
Insufficient laboratory capacity	10 (12.5)	57 (35.0)	54 (22.2)	** < 0.001**
Lack of expertise/training within the AMS team	13 (16.3)	32 (19.6)	45 (18.5)	0.644
Suboptimal use of laboratory services	2 (2.5)	35 (21.5)	37 (15.2)	** < 0.001**
Lack of confidence in the hospital's IPC** processes	4 (5.0)	29 (17.8)	33 (13.6)	0.011
Lack of trust in prescribing guidelines	7 (8.8)	23 (14.1)	30 (12.4)	0.324
Regular shortages/stock outs of essential antibiotics	4 (5.0)	24 (14.7)	28 (11.5)	0.044
Patient demands	7 (8.8)	18 (11.0)	25 (10.3)	0.743
Poor quality of antibiotics	0 (0.0)	15 (9.2)	15 (6.2)	0.003
High cost of antibiotics	0 (0.0)	15 (9.2)	15 (6.2)	0.003
No barriers	5 (6.3)	1 (0.6)	6 (2.5)	0.016

*Statistical significance evaluated using the Pearson’s chi-squared test or Fisher’s exact test. Significance level (α) has been corrected for multiple testing. **Infection prevention and control

Values in boldface indicate statistical significance

## Discussion

### Global-PPS experiences

Since the first Global-PPS in 2015, many hospitals worldwide have collected PPS data on antimicrobial use, however, little is known on the AMS structures and activities in these hospitals and on the impact of Global-PPS on hospital AMS programmes. The results from a survey in 248 hospitals from 74 countries show that the Global-PPS has been very useful in informing and evaluating stewardship activities in many of the participating hospitals. In nearly all of the hospitals that had collected PPS data, participation in the PPS resulted in the identification of targets for AMS programmes, and up to 69.3% stated that at least one of the AMS components or interventions in their institution was driven by Global-PPS findings. One-third of the hospitals conducted at least one follow-up PPS, the majority of which observed improvements in one or more of the indicators evaluated by the PPS. This process of repeated measurements, combined with effective communication of the results to prescribers, contributes to a system of continuous self-monitoring and feedback, a behaviour change strategy essential to the sustainability of the AMS programme [5, 26]. Although several studies have successfully used point prevalence surveys to assess the impact of hospital AMS activities [27–31], there is still a need for guidance in performing AMS studies with a robust yet more complex design, such as controlled interrupted time-series [6, 32].

### AMS structures and interventions

Substantial regional variations were observed in the percentage of hospitals with a formal AMS programme, and in the level of implementation of different components for stewardship. As such, access to local, evidence-based prescribing guidelines ranged from 31.6% in African hospitals to 95.8% in Northern American hospitals. The authors from a worldwide survey on AMS structures, conducted in 2012, reported overall higher results for the existence of treatment guidelines for nearly all regions [10]. However, as our study specifically assessed the presence of local, evidence-based guidelines, this could suggest that in some hospitals guidelines may not be adapted to the local situation. In LMIC’s specifically, development of antibiotic prescribing guidelines is often constrained by limited expertise with the rigorous methods needed to develop guidelines and by a lack of locally-relevant, high-quality evidence to inform guideline development.[9, 33, 34]. The presence of organisational structures, such as AMS committees and hands-on AMS teams, was particularly high in Northern America, where hospitals typically have AMS programmes with a strong focus on accountability and well-established roles for physicians and pharmacists specialised in infectious diseases, both in the AMS committees and in the day-to-day, operational AMS teams [10, 35, 36]. In settings where these resources are not readily available, a possible approach could be to train non-clinically trained staff members, such as pharmacists, to take the lead on implementing hospital AMS activities and coordinating a multidisciplinary AMS team [37, 38].

Education on AMS was mostly targeted at clinicians, although many hospitals also provided education for nurses and pharmacists. The percentage of hospitals educating nurses on AMS was particularly high in our study, and exceeded the percentage of hospitals providing education to pharmacists across all continents. This is in contrast with an international inventory of AMS training programmes performed in 2018, in which AMS training was less likely to be aimed at nurses, compared to other groups of healthcare workers [39].

Our findings suggest that many hospitals were in the process of planning the development of an institutional AMS strategy early 2019, at the time of the survey. It remains to be investigated, however, how the COVID-19 pandemic has impacted the successful development and roll-out of these activities. While the pandemic has led to a disruption of health services worldwide, hospital infectious disease and microbiology teams have been confronted with a shift in priorities, a re-allocation of resources and a reduction of AMS activities [40, 41]. Moreover, in settings where dedicated AMS resources are already scarce, the disruptive impact on hospital AMS activities is expected to be even higher [42].

### Barriers to implementing AMS

As demonstrated in earlier studies[10, 43–47], organisational factors such as a lack of financial and human resources remain important barriers to the implementation of AMS. This could explain why only one-third of hospitals had conducted a follow-up PPS at the time of the survey, as incorporating the PPS throughout the hospital’s AMS activities requires a dedicated and sustainable investment of time and resources. Hospitals in LMIC were more often confronted with unavailability of prescribing guidelines, insufficient laboratory capacity and suboptimal use of the available laboratory services. Indeed, in addition to strengthening the microbiological laboratory capacity in LMIC, efforts should be directed at promoting diagnostic stewardship and improving the communication interface between clinicians and laboratory services [48, 49]. Many hospitals also reported a lack of knowledge on good prescribing practices and a lack of cooperation from prescribers as key barriers to a successful AMS programme. The importance of social and contextual factors determining antimicrobial prescribing behaviour is increasingly being acknowledged. In recent years, qualitative studies have explored some of the drivers behind hospital prescribing practices, such as medical hierarchies and social norms[31, 47, 50, 51], the dynamic between immediate, individual patient care and long-term impact on AMR[31, 52] and clinical uncertainty[51, 53]. Since many of these barriers and driving factors are context-dependent, identifying barriers and strategies to overcome them on a local level should allow hospitals to tailor AMS interventions more precisely and thereby maximise the sustainability of these interventions [54].

### Limitations

The current study had a number of important limitations; first, as the survey was distributed within national and local networks, it was impossible to define the exact response rate and the degree of non-response. However, several strategies to maximise response rates were used, such as involving participants in design and pilot-testing of the survey and sending out reminders at regular intervals. An approximate response rate was estimated at 25%, by identifying the number of hospitals that had collected Global-PPS data at the time the survey was closed, however, the hospitals that stated that they did not yet conduct the Global-PPS were excluded from this estimation. Second, hospitals were not sampled and were therefore entirely self-selecting, which could contribute to selection bias. Third, hospitals in the Global-PPS network (i.e. hospitals that have conducted the PPS or are planning to do so) may have more mature AMS systems compared to other hospitals that are not involved in any kind of stewardship activities. In addition, the majority of hospitals were tertiary hospitals from high- and middle-income countries. As such, primary health services in more rural settings and hospitals in low-income countries were under-represented in the current survey. Investigating the feasibility and relevance of the Global-PPS in these settings is an important area for further research. Next, results on AMS at regional levels are not generalizable to the entire region, as some regions (e.g. Oceania) are clearly under-represented. Furthermore, regional results may be biased towards countries with a large number of responses such as Canada in Northern America (18/24 hospitals) and Nigeria in Africa (18/38 responses). The survey was self-administered and therefore it was impossible to assess the correct interpretation of the survey questions and the accuracy of the responses. Finally, for the multiple choice questions, there was no choice randomization, which might have introduced a certain bias towards the first answer options.

## Conclusions

This study shows substantial variation in hospital AMS programmes and barriers to implementation of AMS across regions and income levels globally. These gaps are likely even larger worldwide than the differences observed here, as the majority of the hospitals participating in the survey were those institutions with time and resources to engage in surveillance activities, highlighting the need for a contextualized approach to antimicrobial resistance in hospitals around the world.

The current study sheds a light on how participation in the Global-PPS can contribute to hospital AMS activities, both in high-income settings and in LMIC. Providing all participating hospitals with a personalised feedback report, the Global-PPS allows local teams to identify targets for antimicrobial stewardship without the need to invest time and resources in complex data analyses. More is still to be gained in guiding hospitals to integrate the PPS throughout AMS activities building on what is already existing, support them in broadly and effectively communicating the PPS results to obtain much-needed buy-in from prescribers, hospital management and other healthcare workers involved in stewardship, and to empower local champions to take the lead on AMS in their hospital. The results presented here will inform the further development of a set of dedicated educational resources, targeting Global-PPS participants worldwide and focused on translating PPS-findings into locally-tailored AMS interventions thus contributing to a sustained response to AMR in participating hospitals.

## Supplementary Information

**Additional file 1.** Global-PPS antimicrobial stewardship survey

**Additional file 2.** Overview of hospitals participating in the antimicrobial stewardship survey, by country and continent.

**Additional file 3.** Hospital characteristics, by region, n hospitals (%).

**Additional file 4.** Antimicrobial stewardship structures in hospitals that conducted the Global-PPS versus hospitals that did not yet conduct the Global-PPS.

## Data Availability

The datasets used and/or analysed during the current study are available from the corresponding author on reasonable request.

## Abbreviations

AMR: Antimicrobial resistance
AMS: Antimicrobial stewardship
IPC: Infection prevention and control
LMIC: Low- and middle-income countries
MDRO: Multi-drug resistant organisms
PPS: Point prevalence survey
