# Supplementary material for: Assessing the impact of the Global Point Prevalence Survey of Antimicrobial Consumption and Resistance (Global-PPS) on hospital antimicrobial stewardship programmes: results of a worldwide survey
Source: Antimicrob Resist Infect Control. 2021 Sep 28;10:138. doi: 10.1186/s13756-021-01010-w (PMC8478001; doi:10.1186/s13756-021-01010-w)
Supplement: Supplementary file 1 — Additional file 1. Global-PPS antimicrobial stewardship survey [file 13756_2021_1010_MOESM1_ESM.pdf]

Demographic data

Please, fill out only one survey per hospital.

1. What is the name of the hospital participating in the Global-PPS?

   Global-PPS hospital ID (optional).

2. In which country is your hospital located?
3. How would you describe your hospital?
   - ☐ Tertiary hospital
   - ☐ Secondary hospital
   - ☐ Primary care institution
   - ☐ Infectious diseases specialized hospital
   - ☐ Paediatric hospital
   - ☐ Other specialized hospital
4. Is your hospital a teaching hospital?
   i.e. a hospital with structured teaching activities
   - ☐ Yes
   - ☐ No
5. How many inpatient beds does your hospital have?
   Inpatient beds: accommodate hospitalized patients who stay in the hospital for a minimum of one night
   - ☐ Less than 100
   - ☐ 101 – 250
   - ☐ 251 – 500
   - ☐ 501-1000
   - ☐ 1001-2000
   - ☐ More than 2000
6. What is your main role in the hospital?
   - ☐ Clinician
   - ☐ Pharmacist
   - ☐ Nurse
   - ☐ Medical microbiologist
   - ☐ Infectious diseases specialist
   - ☐ Infection prevention and control (IPC) specialist
   - ☐ Hospital management
   - ☐ Other, specify:

Global PPS experiences

7. When did your hospital participate in the Global-PPS?
   Multiple answers are possible.
   - ☐ 2015
   - ☐ 2017
   - ☐ 2018
   - ☐ 2019
   - ☐ We did not participate (yet) (go to question 14)

8. Why did your hospital participate in the Global-PPS?
   Multiple answers are possible.
   - ☐ We heard about the Global-PPS from colleagues or via a congress
   - ☐ Multi-drug resistance is an important problem in our hospital
   - ☐ We were asked to participate through a local or national hospital network
   - ☐ We were asked to participate by the ministry of health or other governmental agencies
   - ☐ We were asked to participate by a national or international, non-governmental organization (e.g. MSF)
   - ☐ Other, specify:
   - ☐ Unknown

9. In the following questions we would like to ask your opinion on the **personalized feedback report**. This is the report you can download in pdf-format after validation of your Global-PPS data. Do you use this feedback report?
   - ☐ Yes
   - ☐ No (go to question 13)

10. **Please consider the following 3 statements about the feedback report;**

    a. *"The language & terminology of the feedback report were easy to understand."*
    - ☐ Strongly disagree
    - ☐ Disagree
    - ☐ Neither agree nor disagree
    - ☐ Agree
    - ☐ Strongly agree

b. *“The __scientific content of__ the feedback report was easy to understand.”*

☐ Strongly disagree

☐ Disagree

☐ Neither agree nor disagree

☐ Agree

☐ Strongly agree

c. *“The feedback report was useful.”*

☐ Strongly disagree

☐ Disagree

☐ Neither agree nor disagree

☐ Agree

☐ Strongly agree

11. How useful were the following elements of the feedback report to you?

Every option refers to a specific section in the feedback report.

	Not at all useful	Slightly useful	Moderately useful	Very useful	Extremely useful
**Antimicrobial prevalence by type of ward** (e.g. adult medical wards, neonatal wards, surgical wards, ICU)	○	○	○	○	○
**Proportional antibiotic use**: overall, by antibiotic subgroup or by type of patient	○	○	○	○	○
**Information on quality indicators for antimicrobial use** (e.g. indication in notes, guideline compliance, stop/review date documented…)	○	○	○	○	○
**Top 5 most frequently used antibiotics by indication** (e.g. sepsis, pneumonia, surgical prophylaxis…)	○	○	○	○	○
**Information on duration of prophylaxis** (surgical or medical)	○	○	○	○	○
**Key prescription patterns** (e.g. percentage of IV prescriptions, use of multiple antibiotics)	○	○	○	○	○
**Information on type of antibiotic treatment (empiric vs targeted)** by patient type and by activity	○	○	○	○	○

12.Do you have any suggestions on how to improve the feedback report? (optional free text)

13. According to your first Global-PPS findings, what are/were the most common problems related to antimicrobial use in your hospital?
Multiple answers are possible.

- ☐ High **antimicrobial prevalence rates** (overall or on certain wards)
- ☐ High **proportional use** of a certain class of antibiotics (overall or for a certain type of patient)
- ☐ **Indication for antimicrobial prescription** is not documented in the patient notes
- ☐ Limited or no **availability of prescribing guidelines**
- ☐ Limited or no **compliance to prescribing guidelines**
- ☐ **Stop or review date** of antimicrobial prescriptions is not documented in the patient notes
- ☐ **Inappropriate use** of a certain antimicrobial for a certain indication
- ☐ **Prolonged prophylaxis** (surgical or medical)
- ☐ High amount of **intravenous antibiotic prescriptions**
- ☐ Frequent use of **multiple antibiotics** (per indication / patient)
- ☐ Mainly **empirical antimicrobial use** (antimicrobial use is rarely/never based on microbiological results)
- ☐ Other, specify:
- ☐ Unknown
- ☐ None

Antimicrobial stewardship activities

14. Which of the following **antimicrobial stewardship components** are currently in place in your hospital?

Multiple answers are possible.

- ☐ Development or review of local, evidence-based **guidelines** for antimicrobial prescription
- ☐ Implementation of an antimicrobial **formulary** (i.e. a list of restricted/approved antimicrobials)
- ☐ An active **antimicrobial stewardship committee** (i.e. an organizational structure – stand-alone or embedded in another structure- responsible for defining the antimicrobial stewardship strategy)
- ☐ An active **antimicrobial stewardship team** (i.e. core operational team, responsible for the implementation of the antimicrobial stewardship activities in daily practice)
- ☐ Design of **interventions**, specifically targeted at the antimicrobial prescription (e.g. intravenous-to-oral switch, PKPD dose optimization, automatic stop/review policy, audit and feedback)

  If possible, specify which interventions. (optional)
- ☐ Dedicated **education and communication** to guide antimicrobial prescribing
- ☐ Use of **information technology** to support antimicrobial prescribing (e.g. electronic decision support, mobile phone app)
- ☐ Other, specify:
- ☐ None
- ☐ Unknown

15. Of the stewardship components currently in place in your hospital, please indicate the components that were initiated **as a result of the Global-PPS findings**.

Multiple answers are possible.

- ☐ Development or review of local, evidence-based **guidelines** for antimicrobial prescription
- ☐ Implementation of an antimicrobial **formulary** (i.e. a list of restricted/approved antimicrobials)
- ☐ An active **antimicrobial stewardship committee** (i.e. an organizational structure – stand-alone or embedded in another structure- responsible for defining the antimicrobial stewardship strategy)
- ☐ An active **antimicrobial stewardship team** (i.e. core operational team, responsible for the implementation of the antimicrobial stewardship activities in daily practice)
- ☐ Design of **interventions**, specifically targeted at the antimicrobial prescription (e.g. intravenous-to-oral switch, PKPD dose optimization, automatic stop/review policy, audit and feedback)

  If possible, specify which interventions. (optional)
- ☐ Dedicated **education and communication** to guide antimicrobial prescribing
- ☐ Use of **information technology** to support antimicrobial prescribing (e.g. electronic decision support, mobile phone app)
- ☐ Other, specify:
- ☐ None
- ☐ Unknown

16. Has your hospital organized **<u>educational activities</u>** for healthcare professionals on the topic of antimicrobial stewardship? Indicate for each item which professionals were targeted.

	Clinicians	Nurses	Pharmacists	Other staff
**Written information** *(e.g. leaflets, guideline booklets, newsflashes, "antibiotic of the month"…)*	◯	◯	◯	◯
**Occasional training sessions , ≤ 1 day**	◯	◯	◯	◯
**Regular training sessions, ≤ 1 day** *(e.g. monthly, quarterly…)*	◯	◯	◯	◯
**Occasional courses, > 1 day**	◯	◯	◯	◯
**Regular courses, >1 day** *(e.g. monthly, quarterly…)*	◯	◯	◯	◯
**E-learning**	◯	◯	◯	◯
**Practical, on-the-job training** *(e.g. during ward rounds)*	◯	◯	◯	◯
**Other, specify:**	◯	◯	◯	◯

Impact of antimicrobial stewardship activities

17. Have you performed a **<u>follow-up PPS</u>** to assess the impact of your stewardship activities on antimicrobial use in the hospital?

- ☐ Yes
- ☐ No (go to question 19)
- ☐ Planned (go to question 19)
- ☐ I don't know (go to question 19)

18. a. In the results of your follow-up PPS, have you observed any of the following trends?

Multiple answers are possible.

- ☐ A decrease in **antimicrobial prevalence rates** (overall or on certain wards)
- ☐ A decrease in the use of a **certain class of antibiotics** (overall or for a certain type of patient)
- ☐ **Indication for antimicrobial prescription** is documented more often (overall or for a certain ward type)
- ☐ Increased **availability of prescribing guidelines** (overall or for a certain ward type)
- ☐ Increased **compliance to prescribing guidelines** (overall or for a certain ward type)
- ☐ **Stop or review date of antimicrobial prescriptions** is documented more often (overall or for a certain ward type)
- ☐ Shorter **duration of prophylaxis** (surgical or medical)
- ☐ A decrease in the amount of **intravenous antibiotic prescriptions**
- ☐ A decrease in the use of **multiple antibiotics** (per indication / patient)
- ☐ An increase in the amount of **targeted prescriptions** (based on microbiology results)
- ☐ Other, specify:
- ☐ None
- ☐ Unknown

b. Were there any external factors that could have influenced this result, e.g. seasonal variation, change in staffing levels, hospital reorganization and restructuring? (free, optional text field)

Barriers

19. a. What are the **main barriers** to implement an effective antimicrobial stewardship program in your hospital?
Please select maximum 5 answers.

- ☐ Lack of qualified personnel
- ☐ Qualified personnel does not have enough time to perform stewardship
- ☐ Lack of funding
- ☐ Lack of support from hospital management
- ☐ Insufficient microbiology laboratory capacity
- ☐ Inadequate use of the microbiology laboratory
- ☐ Poor quality of antibiotics
- ☐ Regular shortages or stock outs of essential antibiotics
- ☐ High cost of antibiotics
- ☐ Lack or unavailability of practical, evidence-based, local guidelines
- ☐ Lack of trust in local guidelines
- ☐ Lack of cooperation from prescribers
- ☐ Lack of knowledge on good prescribing practices among clinicians
- ☐ Lack of expertise and training in antimicrobial stewardship within the antimicrobial stewardship team
- ☐ Lack of confidence in the hospital infection prevention and control (IPC) processes
- ☐ Lack of information technology support
- ☐ Patient demands or beliefs
- ☐ Other, specify
- ☐ No barriers

b. Could you **rank the barriers** you selected according to how important they are?
Drag and drop the barriers in the correct sorting order, where "1" is the most important barrier.

Future plans for antimicrobial stewardship

20. Does your hospital have a **formal antimicrobial stewardship strategy** (i.e. a plan that describes the aims, milestones and outcome measures of stewardship activities in your hospital)?

- ☐ Yes
- ☐ No
- ☐ Planned
- ☐ I don't know

21. What would be the main motivations to perform repeated PPSs of antimicrobial use in your hospital?
Please select maximum 3 answers.

- ☐ It creates awareness on appropriate antimicrobial prescribing among prescribers
- ☐ It allows us to be part of a local or national network of hospitals
- ☐ It convinces our hospital management to keep investing resources in antimicrobial stewardship
- ☐ It allows us to continuously monitor the quality and quantity of antimicrobial prescriptions
- ☐ It allows us to measure the impact of our antimicrobial stewardship activities
- ☐ Other, specify:
- ☐ We will not perform a repeated PPS

22. According to you, what would be the feasible frequency for repeated PPSs of antimicrobial use in your hospital in the future?

	2-3 times/year	Every year	Every 2 years	Occasionally	None
All wards	○	○	○	○	○
ICU wards	○	○	○	○	○
Medical wards	○	○	○	○	○
Surgical wards	○	○	○	○	○
Other, specify:	○	○	○	○	○

23. Please indicate which of the following **educational activities** on antimicrobial stewardship you think would support your hospital in continuing its stewardship efforts.
Multiple answers are possible.

- ☐ Face-to-face training sessions from stewardship champions
- ☐ Online learning solutions (e-learning)
- ☐ Interactive discussion forum
- ☐ Joint research activities (stewardship implementation research in collaboration with another institute)
- ☐ Case-based learning (real-world examples)
- ☐ Other, specify:
- ☐ None

24. Please indicate which of the **educational topics** you think would support your hospital in continuing antimicrobial stewardship efforts.
Please select maximum 5 answers.

- ☐ "What is the low-hanging fruit for antimicrobial stewardship in my hospital?"
  i.e. easy and effective antimicrobial stewardship interventions
- ☐ "How to optimize therapeutic antimicrobial use?"
- ☐ "How to optimize surgical prophylaxis?"
- ☐ "How to formulate or revise a guideline?"
- ☐ "How to perform audit and feedback?"
- ☐ "How to create an active stewardship committee/team?"
- ☐ "How to translate your PPS results into stewardship interventions?"
- ☐ "How to translate your PPS results into infection prevention and control (IPC) interventions?"
- ☐ "How to manage infections caused by difficult-to-treat MDRO (multidrug-resistant organisms)?"
- ☐ "How to understand antimicrobial susceptibility data?"
- ☐ "How to communicate with prescribers on antimicrobial prescribing?"
- ☐ "How to communicate with patients on antimicrobial use?"
- ☐ Other, specify:
- ☐ None
