# Supplementary material for: Assessing the impact of the Global Point Prevalence Survey of Antimicrobial Consumption and Resistance (Global-PPS) on hospital antimicrobial stewardship programmes: results of a worldwide survey
Source: Antimicrob Resist Infect Control. 2021 Sep 28;10:138. doi: 10.1186/s13756-021-01010-w (PMC8478001; doi:10.1186/s13756-021-01010-w)
Supplement: Supplementary file 2 — Additional file 2. Overview of hospitals participating in the antimicrobial stewardship survey, by country and continent. [file 13756_2021_1010_MOESM2_ESM.pdf]

Additional file 2. Overview of hospitals participating in the antimicrobial stewardship survey, by country and continent

Region	Country	Hospitals planning to conduct PPS	Hospitals that conducted PPS	Total number of hospitals
**Africa**		**15**	**23**	**38**
	Cameroon		2	2
	Egypt		2	2
	Gambia		1	1
	Ghana	1	1	2
	Guinea		1	1
	Kenya	3		3
	Liberia	2	1	3
	Mauritania	1		1
	Nigeria	5	13	18
	South Africa	1		1
	Tanzania	1		1
	Tunisia		1	1
	Uganda	1	1	2
**Asia**		**14**	**77**	**91**
	Bahrain		1	1
	Cambodia		1	1
	China	1	2	3
	Georgia		18	18
	India	5	11	16
	Iran	1	3	4
	Israel		2	2
	Japan		5	5
	Jordan	1		1
	Republic of Korea		1	1
	Kuwait		1	1
	Kyrgyzstan		3	3
	Laos		1	1
	Lebanon		1	1
	Malaysia	1		1
	Pakistan		1	1
	Philippines		16	16
	Saudi Arabia		3	3
	Singapore		3	3
	Taiwan	1		1
	Thailand	1	1	2
	Turkey	2	1	3
	United Arab Emirates	1		1
	Vietnam		1	1
	Yemen		1	1

Region	Country	Hospitals planning to conduct PPS	Hospitals that conducted PPS	Total number of hospitals
**Europe**		**11**	**50**	**61**
	Albania		2	2
	Belgium		1	1
	Bosnia and Herzegovina	1	1	2
	Bulgaria		1	1
	Croatia		3	3
	France	2	2	4
	Germany		1	1
	Greece		2	2
	Hungary	1	1	2
	Italy	2	5	7
	Kosovo*		1	1
	Lithuania		1	1
	Montenegro	3	2	5
	Netherlands		1	1
	Portugal		1	1
	Romania	1		1
	Russia		13	13
	Serbia		3	3
	Slovenia		1	1
	Spain		3	3
	Sweden		1	1
	Ukraine		1	1
	United Kingdom	1	3	4
**Oceania**		**1**	**2**	**3**
	Australia		2	2
	New Zealand	1		1
**Northern America**		**7**	**17**	**24**
	Canada	2	16	18
	USA	5	1	6
**Latin America - Caribbean**		**8**	**23**	**31**
	Argentina	1	2	3
	Bolivia	1		1
	Brazil	3	6	9
	Chile		4	4
	Colombia	1	1	2
	Costa Rica		2	2
	Mexico		8	8
	Trinidad And Tobago	1		1
	Venezuela	1		1
**Grand Total**		**56**	**192**	**248**

*In accordance with Security Council Resolution 1244 (1999)
