# Supplementary material for: Assessing the impact of the Global Point Prevalence Survey of Antimicrobial Consumption and Resistance (Global-PPS) on hospital antimicrobial stewardship programmes: results of a worldwide survey
Source: Antimicrob Resist Infect Control. 2021 Sep 28;10:138. doi: 10.1186/s13756-021-01010-w (PMC8478001; doi:10.1186/s13756-021-01010-w)
Supplement: Supplementary file 3 — Additional file 3. Hospital characteristics, by region, n hospitals (%). [file 13756_2021_1010_MOESM3_ESM.pdf]

Additional file 3. Hospital characteristics, by region, n hospitals (%)

	Hospitals that conducted PPS (n = 192)						Hospitals planning to conduct PPS (n = 56)					
	**Africa**	**Asia**	**Europe**	**Latin America - Caribbean**	**Northern America**	**Oceania**	**Africa**	**Asia**	**Europe**	**Latin America - Caribbean**	**Northern America**	**Oceania**
**Hospital type***												
Tertiary hospital	18 (78.3)	55 (71.4)	31 (62.0)	20 (87.0)	8 (47.1)	2 (100)	5 (33.3)	9 (64.3)	5 (45.5)	7 (87.5)	3 (42.9)	
Secondary hospital	3 (13.0)	8 (10.4)	10 (20.0)	1 (4.3)	6 (35.3)		8 (53.3)	2 (14.3)	3 (27.3)	1 (12.5)	1 (14.3)	1 (100)
Paediatric hospital		7 (9.1)	4 (8.0)		1 (5.9)				1 (9.1)			
Other specialised hospital	1 (4.3)	5 (6.5)	1 (2.0)				1 (6.7)	2 (14.3)	1 (9.1)		1 (14.3)	
Primary care institution			3 (6.0)	1 (4.3)	2 (11.8)		1 (6.7)	1 (7.1)	1 (9.1)		2 (28.6)	
Infectious diseases specialised hospital	1 (4.3)	2 (2.6)	1 (2.0)	1 (4.3)								
**Teaching hospital**												
Yes	18 (78.3)	54 (70.1)	49 (98.0)	18 (78.3)	13 (76.5)	1 (50.0)	13 (86.7)	9 (64.3)	8 (72.7)	6 (75.0)	7 (100)	1 (100)
No	5 (21.7)	23 (29.9)	1 (2.0)	5 (21.7)	4 (23.5)	1 (50.0)	2 (13.3)	5 (35.7)	3 (27.3)	2 (25.0)		
**Number of inpatient beds****												
Less than 100	3 (13.0)	13 (16.9)		3 (13.0)	1 (5.9)		1 (6.7)	2 (14.3)	1 (9.1)	2 (25.0)	2 (28.6)	
101 – 250	4 (17.4)	18 (23.4)	7 (14.0)	10 (43.5)	4 (23.5)		5 (33.3)	3 (21.4)	3 (27.3)	2 (25.0)	1 (14.3)	
251 – 500	7 (30.4)	19 (24.7)	14 (28.0)	9 (39.1)	8 (47.1)	2 (100)	5 (33.3)	3 (21.4)	3 (27.3)	4 (50.0)	1 (14.3)	1 (100)
501 - 1000	7 (30.4)	15 (19.5)	17 (34.0)		4 (23.5)		3 (20.0)	2 (14.3)	2 (18.2)		3 (42.9)	
1001 - 2000	1 (4.3)	9 (11.7)	9 (18.0)	1 (4.3)				3 (21.4)	2 (18.2)			
More than 2000	1 (4.3)	3 (3.9)	3 (6.0)				1 (6.7)	1 (7.1)				

* Tertiary hospital: clinical services are highly differentiated by function. Provides regional services and regularly takes referrals from other (primary and secondary) hospitals. Secondary hospital: clinical services are highly differentiated by function. Takes some referrals from other (primary) hospitals. Primary care institution: has only few medical specialties. Only limited laboratory services are available. Infectious diseases specialised hospital and paediatric hospital: single clinical specialty, possibly with sub-specialties. Highly specialised staff and technical equipment.** Inpatient beds: accommodate hospitalized patients who stay in the hospital for a minimum of one night.
