# Supplementary material for: Assessing the impact of the Global Point Prevalence Survey of Antimicrobial Consumption and Resistance (Global-PPS) on hospital antimicrobial stewardship programmes: results of a worldwide survey
Source: Antimicrob Resist Infect Control. 2021 Sep 28;10:138. doi: 10.1186/s13756-021-01010-w (PMC8478001; doi:10.1186/s13756-021-01010-w)
Supplement: Supplementary file 4 — Additional file 4. Antimicrobial stewardship structures in hospitals that conducted the Global-PPS versus hospitals that did not yet conduct the Global-PPS. [file 13756_2021_1010_MOESM4_ESM.pdf]

Additional file 4. Antimicrobial stewardship structures in hospitals that conducted the Global-PPS versus hospitals that did not yet conduct the Global-PPS

	n (%)			
	**Hospitals planning to conduct PPS (n = 56)**	**Hospitals that conducted PPS (n = 192)**	**Total number of hospitals (n = 248)**	**P-value* (α=0.005)**
Local, evidence-based guidelines	32 (57.1)	143 (74.5)	**175 (70.6)**	0.019
Antimicrobial formulary	30 (53.6)	126 (65.6)	**156 (62.9)**	0.137
AMS committee**	25 (44.6)	102 (53.1)	**127 (51.2)**	0.334
AMS team†	24 (42.9)	95 (49.5)	**119 (48.0)**	0.471
Specific AMS interventions††	18 (32.1)	81 (42.2)	**99 (39.9)**	0.232
Education and communication	31 (55.4)	98 (51.0)	**129 (52.0)**	0.677
Information technology support	14 (25.0)	62 (32.3)	**76 (30.6)**	0.381
Other AMS activities	3 (5.4)	7 (3.7)	**10 (4.0)**	0.699
No AMS activities	9 (16.1)	11 (5.7)	**20 (8.1)**	0.022

* Statistical significance evaluated using the Pearson's chi-squared test or Fisher's exact test. Significance level (α) has been corrected for multiple testing.

** the organizational structure responsible for defining the antimicrobial stewardship strategy.

† the core operational team, responsible for the implementation of the antimicrobial stewardship activities in daily practice.

†† e.g. audit and feedback, automatic stop orders, intravenous-to-oral switch policies etc…
